# Comparative Analysis of Co-Cultured Amniotic Cell-Conditioned Media with Cell-Free Amniotic Fluid Reveals Differential Effects on Epithelial–Mesenchymal Transition and Myofibroblast Activation

**DOI:** 10.3390/biomedicines10092189

**Published:** 2022-09-05

**Authors:** Naiyou Liu, Charles M. Bowen, Mohammadali M. Shoja, Karen Larissa Castro de Pereira, Laxmi Priya Dongur, Antonio Saad, William K. Russell, Thomas Christopher Broderick, Jeffrey H. Fair, William Samuel Fagg

**Affiliations:** 1 Division of Transplant, Department of Surgery, University of Texas Medical Branch, Galveston, TX 77555, USA; 2 John Sealy School of Medicine, University of Texas Medical Branch, Galveston, TX 77555, USA; 3 Department of Obstetrics and Gynecology, University of Texas Medical Branch, Galveston, TX 77555, USA; 4 Department of Biochemistry and Molecular Biology, University of Texas Medical Branch, Galveston, TX 77555, USA; 5 Merakris Therapeutics, RTP Frontier, Research Triangle Park, NC 27709, USA; 6 Golden LEAF Biomanufacturing Training and Education Center, North Carolina State University, Raleigh, NC 27606, USA

**Keywords:** myofibroblast, amniotic fluid, regenerative medicine, epithelial–mesenchymal transition, stem cells

## Abstract

Myofibroblast activation is a cellular response elicited by a variety of physiological or pathological insults whereby cells initiate a coordinated response intended to eradicate the insult and then revert back to a basal state. However, an underlying theme in various disease states is persistent myofibroblast activation that fails to resolve. Based on multiple observations, we hypothesized that the secreted factors harvested from co-culturing amniotic stem cells might mimic the anti-inflammatory state that cell-free amniotic fluid (AF) elicits. We optimized an amnion epithelial and amniotic fluid cell co-culture system, and tested this hypothesis in the context of myofibroblast activation. However, we discovered that co-cultured amniotic cell conditioned media (coACCM) and AF have opposing effects on myofibroblast activation: coACCM activates the epithelial–mesenchymal transition (EMT) and stimulates gene expression patterns associated with myofibroblast activation, while AF does the opposite. Intriguingly, extracellular vesicles (EVs) purified from AF are necessary and sufficient to activate EMT and inflammatory gene expression patterns, while the EV-depleted AF potently represses these responses. In summary, these data indicate that coACCM stimulates myofibroblast activation, while AF represses it. We interpret these findings to suggest that coACCM, AF, and fractionated AF represent unique biologics that elicit different cellular responses that are correlated with a wide variety of pathological states, and therefore could have broad utility in the clinic and the lab.

## 1. Introduction

Cells respond to external inputs in a manner that dictates how they, and thus the overall tissue, respond to a stimulus. In the contexts of physical, mechanical, environmental, or metabolic damage these cellular responses can include myofibroblast activation (MFA) [1,2]. This is a complex response in which the resident cell types within the tissue of interest undergo a transformation into a contractile phenotype and then remodeling and secretion of extracellular matrix components [2]. An underlying theme in various pathologies is that unchecked MFA can lead to chronic inflammation and fibrosis that may eventually lead to organ failure. Specifically the underlying molecular and cellular pathologies in patients suffering from chronic wounds [3], and liver [4], kidney [5], pancreas [6], or heart fibrosis/failure [7] indicate that unresolved MFA drives the persistent inflammatory state [8,9]. Fortunately, modeling MFA in vitro is relatively straightforward, and is preceded by the epithelial–mesenchymal transition (EMT), another complex process that occurs normally during development, but contributes to various pathologies when unchecked [10,11,12,13,14]. Thus, assays to monitor EMT/MFA consist of measuring cell migration and gene expression profiling for biomarkers of the EMT [15] and upregulation of classic fibrosis-associated genes such as smooth muscle actin (*Acta2*) and type I collagen (*Col1a1*), amongst others [16,17]. These observations indicate that reducing unchecked EMT and MFA represent a potential therapeutic opportunity [18] in which discovery-stage reagents can be readily tested in vitro.

Cell-free AF (cfAF, herein referred to as AF for the remainder) is safe and appears to be effective in treating wounds, osteoarthritis, and other degenerative conditions in both the clinic [19,20,21,22,23,24,25,26] and research lab [27,28,29,30,31,32,33,34,35,36,37,38,39,40,41,42,43,44,45]. It is clear that this complex biofluid contains proteins with chemotactic, pro-growth, and anti-inflammatory properties [46,47,48], but open questions remain regarding its use in regenerative medicine. For example, the cellular, molecular, and biochemical mechanisms through which AF executes its therapeutic functions are ill-defined, and so its full potential in this context is unknown. Furthermore, AF donor-to-donor variability is the source of fluctuating levels of total protein, specific cytokines/chemokines, extracellular vesicles (EVs), and other biomolecules [46,49,50,51,52]. These dissimilarities in the source material could lead to inconsistent outcomes, with further variation depending on the desired therapeutic context(s) and patient population(s). Thus, two major unmet needs are to illuminate the mechanisms through which AF stimulates healing and to develop standardized methods to either validate, or consistently manufacture, AF-like substances with defined levels of discrete components.

Multipotent progenitor cells derived from full-term birth tissues such as amnion epithelial cells (AECs) and amniotic fluid cells (AFCs) have also shown diverse therapeutic potential. These have been more extensively studied than AF, so many excellent reviews are available [53,54,55,56,57,58]. These bodies of literature suggest that AECs and AFCs have the potential to effectively treat a variety of conditions, ranging from cutaneous wounds to orthopedic conditions, to liver or lung disorders, and neurological conditions. While some efficacy has been shown in certain clinical contexts [59,60,61], it is clear that these treatments are safe when properly administered [62,63]. Furthermore, the therapeutic effects appear to be mediated not necessarily by the cells directly, but by their secreted trophic factors [64,65,66,67,68]. Similar to AF, the components found in AEC and AFC secretomes and the effects they stimulate are primarily anti-inflammatory, immunomodulatory, and pro-regenerative. Given these and other observations, AEC- and AFC-secreted factors are attractive candidates to reduce EMT and MFA.

We hypothesized that the use of AF and/or its stem cell and secretory factors might fill the need for broadly applicable approaches to reduce MFA. We first optimized a co-cultured Amniotic Cell Conditioned Media (coACCM) system which enabled us to consistently harvest maximal levels of secreted factors. We compared the coACCM to AF, under the presumption that they would elicit similar cellular responses in myoblasts and fibroblasts, but found that they actually promote opposing effects. Specifically, the coACCM promotes EMT and gene expression patterns associated with MFA while AF repressed these. Moreover, fractionation of coACCM into purified EVs or coACCM depleted of EVs had little effect on these cellular responses compared to total coACCM. In contrast, AF EVs were necessary and sufficient to promote EMT and MFA, while EV-depleted AF potently repressed these processes. We interpret these results to indicate that coACCM, AF EVs, total AF, and EV-depleted AF have distinct effects on EMT and MFA and may represent novel solutions that can be used for phased responses in precision regenerative medicine.

## 2. Materials and Methods

### 2.1. Cell Culture

**Figure 1:**
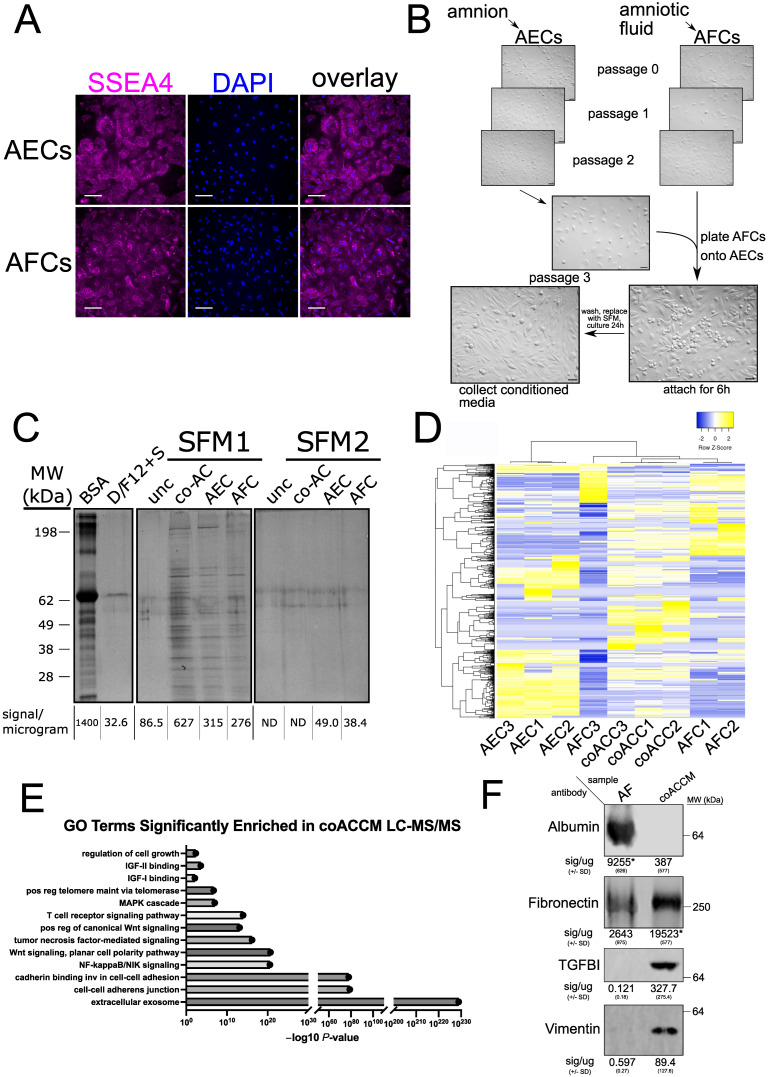
**An optimized co-culture system to generate a robust secreted proteome.** (**A**) Indirect immunofluorescence using stage-specific antigen 4 (SSEA4) antibody (magenta, *left*), DAPI (blue, *middle*) and channels overlaid (*right*). On the top panel, AECs and on the bottom panel AFCs are scale bar = 100 µm. (**B**) Experimental model of conditioned media production. AECs were isolated from amnion, while AFCs were isolated from AF. After the third cell passage, AECs were mitotically inactivated by mitomycin treatment, plated and allowed to attach. Then, AFCs were plated with AECs and allowed to attach for 6 h, then serum-containing media was removed, and serum-free media added. Conditioned media was collected 24 h later. (**C**) SDS-PAGE and silver staining analysis of 1 µg of BSA, DMEM/F12 + 10% FBS (D/F12 + S; 10 µg), unconditioned media (unc), co-cultured amniotic cell (coAC; 10 µg), AEC-only conditioned media (AEC; 10 µg), and AFC-only conditioned media (AFC; 10 µg); molecular weight (MW) in kilodaltons (kDa) is shown on the left. (**D**) Clustered heatmap of proteins measured in SFM, coACCM, AEC-only and AFC-only conditioned media by LC-MS/MS. (**E**) Gene ontology analysis of the parent genes associated with cellular components, molecular functions, or biological processes that encode the proteins/peptides identified in LC-MS/MS coACCM compared to unconditioned SFM. (**F**) Representative Western blot (see also Supplementary Figure S1) of 10 µg of protein from AF or coAACM probed with antibodies for Albumin, Fibronectin, TGFb induced protein and Vimentin; the mean numerical signal per microgram protein is shown below +/− SD (* *p* < 0.05 by Student’s *t*-test); MW in kDa is shown on the right.

C2C12 myoblasts were cultured as previously described [69] in DMEM + 10% FBS, as were MMM fibroblasts [70]. AECs were either purchased from ScienCell Research Laboratories (Cat. # 7110) or detached from amnion from donors undergoing elective Cesarean delivery as previously described [71], and cultured in (DMEM/F12 with 10% FBS, 1.05 mM Ca^2+^, 10 ng/mL EGF and 1% penicillin/streptomycin) on plates coated with type IV collagen. Media were changed every 3 days and cells were passed at a 1:3 ratio with 0.25% Trypsin-EDTA. AFCs were procured from AF donors undergoing elective Cesarean deliveries, and were collected by centrifuging AF at 1200 RPM for 5 min at 4 °C to pellet cells. These were then plated into dishes containing DMEM + 10% FBS + 10% AF and the media was changed every 3 days. Typically, outgrowth of cells was observed after 7–10 days and cells were passed at a 1:3 or 1:5 ratio using 0.25% Trypsin-EDTA. AFC and AEC co-culture was performed on type IV collagen-coated cell culture dishes; briefly, AECs were mitotically inactivated by treatment with 2 µg/mL mitomycin C for 2–3 h, then 20,000 cells per cm^2^ were plated and allowed to attach for 4 h to overnight. AFCs were then plated onto the dish containing AECs at a density of 66,500 cells per cm^2^ and allowed to attached for 4 h. Media was changed to IMDM/F12 (50% each) with 1% polyvinyl alcohol, 1% lipid concentrate (Gibco, Waltham, MA, USA), and 450 µm monothioglycerol (Sigma, Burlington, MA, USA) (serum-free media 1 or SFM1) or DMEM (SFM2) to test optimal serum-free media formulations (see Figure 1) after 24 h of culture. After that, SFM1 was used to generate coACCM; likewise, SFM1 was used as the basal media for subsequent experiments with myoblasts and fibroblasts (except where indicated that DMEM + 10% FBS was used as a control condition).

### 2.2. Amniotic Fluid Collection and Processing

Amniotic fluid was collected from full-term pregnant individuals that screened negative for infectious disease and gestational diabetes, were non-smokers, and were otherwise healthy who were undergoing elective cesarean delivery. We sought IRB approval for AF collection, but the requirement for such was waived, as AF is considered medical waste, and donors from which we procured it were de-identified. The obstetrician used a red Robinson tube connected to a 60 mL syringe to carefully aspirate AF after hysterotomy was performed, and before delivery of the fetus.

The AF was stored on ice and transported to the research lab, then was passed through a 100 µm cell strainer (Falcon; Corning, Inc., Corning, NY, USA) to remove large debris and vernix. It was then centrifuged at 1200 RPM at 4 °C to pellet cells (see above), then centrifuged again at 4000 RPM for 15 min at 4 °C, and then several times at 8000 RPM for 30 min at 4 °C to remove additional insoluble material. It was then further processed by either passing through a resin-bonded glass fiber composite media 0.45 µm depth filter (Pall, Port Washington, NY, USA) for primary clarification followed by a polyethersulfone 0.22 µm (Pall) sterilizing-grade filter.

### 2.3. Extracellular Vesicle Purification and Analysis

EVs were purified from coACCM and AF using the ExoQuick TC-ULTRA kit (System Bio, Palo Alto, CA, USA) or AF EVs were purified by ultracentrifugation with a Beckman XL-80 Ultracentrifuge (Beckman Coulter, Brea, CA, USA) and a SW41TI rotor by 107,000× *g* spin for 90 min at 4 °C. The resulting supernatant was collected as the EV-depleted fraction, and the pellet was washed twice with cold PBS, then resuspended in cold PBS and immediately stored at −80 °C or on ice then further analyzed. Qualitative and quantitative analysis of EVs was performed using nanoparticle tracking analysis (NTA) with the ZetaView particle analyzer model PMX-120 (Particle Metrix, Inning am Ammersee, Germany), transmission electron microscopy (see below), the Bradford method as described previously [72] (BioRad, Hercules, CA, USA), Western blotting (see below), and LC-MS/MS (see below).

For TEM either total AF, purified EVs from AF, or EV-depleted AF were applied to electron microscopic 200 mesh copper grids and negatively stained with 2% aqueous uranyl acetate. They were examined and measured in a JEOL JEM-1400 electron microscope at 80 kV. Images were acquired on bottom-mounted CCD camera Orius SC200 (Gatan, Pleasanton, CA, USA), and were used to measure the size, relative numbers, and shape of EVs.

### 2.4. Liquid Chromatography with Tandem Mass Spectrometry

Sample digestion: approximately 10 µg of sample was diluted to 1 µg/µL in 50 mM Ammonium Bicarbonate, 10 mM DTT at pH = 7.6 and incubated at 95 °C for 10 min. The sample was then cooled to RT and 3.75 µL of 1 M iodoacetamide acid added and allowed to react for 20 min in the dark after which 0.5 µL of 2 M DTT was added to quench the reaction. Trypsin was added to the protein mixture in a ratio of 1:100, vortexed and incubated at 37 °C overnight. Trifluoracetic acid was then added to a final concentration of 0.1% TFA to quench trypsinolysis.

NanoLC MS/MS Analysis: peptide mixtures were analyzed by nanoflow liquid chromatography-tandem mass spectrometry (nanoLC-MS/MS) using a nano-LC chromatography system (UltiMate 3000 RSLCnano, Dionex, Sunnyvale, CA, USA), coupled on-line to a Thermo Orbitrap Fusion mass spectrometer (Thermo Fisher Scientific, San Jose, CA, USA) through a nanospray ion source (Thermo Scientific). A trap and elute method was used. The trap column was a C18 PepMap100 (300 µm × 5 mm, 5 µm particle size) from Thermo Scientific. The analytical column was an Acclaim PepMap 100 (75 µm × 25 cm) from (Thermo Scientific). After equilibrating the column in 98% solvent A (0.1% formic acid in water) and 2% solvent B (0.1% formic acid in acetonitrile (ACN)), the samples (1 µL in solvent A) were injected onto the trap column and subsequently eluted (400 nL/min) by gradient elution onto the C18 column as follows: isocratic at 2% B, 0–5 min; 2% to 32% B, 2–69 min; 32% to 70% B, 69–79 min; 70% to 90% B, in one minute min isocratic at 90% B, 80–84 min; 90% to 2%, 84–85 min; and isocratic at 2% B, 85–90 min.

All LC-MS/MS data were acquired using XCalibur, version 2.1.0 (Thermo Fisher Scientific) in positive ion mode using a top speed data-dependent acquisition (DDA) method with a 3 s cycle time. The survey scans (*m*/*z* 370–1570) were acquired in the Orbitrap at 50,000 resolution (at *m*/*z* = 400) in profile mode, with a maximum injection time of 56 ms and an AGC target of 400,000 ions. The S-lens RF level was set to 60. Isolation was performed in the quadrupole with a 1.6 Da isolation window, and HCD MS/MS acquisition was performed in centroid mode using rapid scan rate with detection in the ion trap, with the following settings: collision energy = 32%; maximum injection time 60 ms; AGC target 200,000 ions. Monoisotopic precursor selection (MIPS) and charge state filtering were on, with charge states 2–6 included. Dynamic exclusion was used to remove selected precursor ions, with a +/− 10 ppm mass tolerance, for 15 s after acquisition of one MS/MS spectrum.

Database Searching: tandem mass spectra were extracted and charge state deconvoluted by Proteome Discoverer (Thermo Fisher, version 1.4.1.14). Deisotoping was not performed. All MS/MS spectra were searched against a Uniprot Human database (version 6 November 2019) using Sequest. Searches were performed with a parent ion tolerance of 5 ppm and a fragment ion tolerance of 0.60 Da. Trypsin was specified as the enzyme, allowing for two missed cleavages. Fixed modification of carbamidomethyl (C) and variable modifications of oxidation (M). Scaffold (version Scaffold_4.8.7, Proteome Software Inc., Portland, OR, USA) was used to validate MS/MS based peptide and protein identifications. Peptide identifications were accepted if they could be established at greater than 95.0% probability. Peptide Probabilities from X! Tandem and Sequest were assigned by the Scaffold Local FDR algorithm. Peptide Probabilities were assigned by the Peptide Prophet algorithm [73] with Scaffold delta-mass correction. Protein identifications were accepted if they could be established at greater than 95.0% probability and contained at least 2 identified peptides. Protein probabilities were assigned by the Protein Prophet algorithm [74]. Proteins that contained similar peptides and could not be differentiated based on MS/MS analysis alone were grouped to satisfy the principles of parsimony.

### 2.5. Microscopy and Analysis

Myoblasts or fibroblasts were added into the appropriate number of wells containing DMEM + 10% FBS in a 24- or 96-well plates one day prior to the experimental procedure such that the cells were approximately 60–80% confluent at the beginning of the experiment. Prior to beginning an experimental procedure or time course, the media were changed to the relevant type, and allowed to equilibrate for two to four hours.

Scratch test cell migration assays were carried out on cells plated in biological triplicate in 24-well plates by implementing a manual scratch down the middle of each well using a P200 pipet tip. Phase contrast images were collected using an IV71 inverted microscope (Olympus, Tokyo, Japan) or the CellAssist automated, temperature and CO_2_ controlled microscopy system (Thrive Bioscience, Wakefield, MA, USA) with images collected at the times indicated. For manually collected images using the Olympus IV71 microscope, for each biological replicate two photographs were collected representing two independent fields of view: one above, and one below, a constant region of interest. The scratch area was determined by measuring its size in pixels via tracing the area(s) devoid of cells and then calculating its size(s). For each biological replicate, these were then averaged, which gave three average sizes of scratch per biological replicate, per timepoint and condition. For images collected with the CellAssist (Thrive Bioscience), a single field of view was analyzed throughout. The scratch areas were then normalized to that recorded at time zero, and the mean values and standard deviations were plotted in Prism 9 (GraphPad Software, San Diego, CA, USA). Statistical significance of each timepoint was determined by using the two-way ANOVA test with multiple comparisons to compare each condition to each other condition.

Quantitative high content imaging was performed using the Opera Phenix High Content Screening System (Perkin Elmer, Waltham, MA, USA), and images were collected using non-confocal mode with the 10X objective. Cells were stained with rabbit anti-Vimentin antibody (abcam ab92547), phalloidin:488 (Thermo Fisher), and DAPI (Thermo Fisher). Image analysis was performed using Harmony software (Perkin Elmer). Cells were plated as described above but in optical 96-well plates (Nunc, Roskilde, Denmark). For measurements calculated per cell, mean fluorescent signal intensities for 500 cells from 5 independent fields of view were determined for one well, then the same was performed for a second independent well, and the resulting values from 1000 cells (representing 10 independent fields of view from 2 independent wells; with the exception of the SFM condition at 24 h for Vimentin and phalloidin, due to a technical error data were only collected from a single well spanning 5 fields of view but still represent 1000 cells) per condition were plotted in Prism 9. Since a normal distribution for these data could not be assumed, statistical significance was tested via the Kruskal–Wallis test with each possible pairwise comparison performed (we also note that we observed similar results using the ordinary one-way ANOVA test with multiple comparisons).

### 2.6. RNA Extractions and RT-qPCR

RNA was extracted from cells as described previously [75] using a ReliaPrep RNA extraction kit (Promega, Madison, WI, USA). RNA yields were measured on a NanoDrop (Thermo Scientific) and between 100–500 ng of RNA was used for reverse transcription using the SuperScript III system (Thermo Scientific) according to the manufacturer’s instructions. The resulting cDNA was diluted between 1:10 or 1:25 in nuclease-free water and one microliter was used as template in a 25 microliter qPCR in which PowerUP SYBR Green Master Mix (Thermo Fisher) master mix was used. Amplification and analysis were performed on a StepOne Applied Biosystems qPCR machine with StepOne software (Applied Biosystems, Waltham, MA, USA). Amplification specificity was confirmed using melt curve analysis. Data were analyzed using the delta-delta Ct method, as described previously [76]. In the event that no amplification was observed, a Ct value of 40 was assigned. Statistical significance between samples was measured by comparing dCt values using either the Student’s *t*-test or the one-way ANOVA test and this is noted in the figure legends.

### 2.7. Protein Sample Preparation, SDS-PAGE, and Western Blotting

Protein concentration was measured using the Bradford method as described previously [72] (BioRad). Samples were prepared for SDS-PAGE by the addition of Laemmli sample buffer and heating to 95 °C for 5 min then a short incubation on ice. Equal amounts of protein were loaded per lane on 10% SDS-PAGE and electrophoresed until the dye front reached the bottom of the gel. For silver staining, gels were removed and stained using the Silver Stain Plus kit (BioRad) according to the manufacturer’s instructions, then the gel was photographed wet, then dried on a gel dryer for longer term storage. Western blotting was performed essentially as described previously [77]: proteins were separated by SDS-PAGE were transferred to nitrocellulose (BioRad) using a wet tris-glycine buffered system (Hoefer, Holliston, MA, USA) for at 100 V (constant voltage) for 90 min at 4 °C. The membrane was blocked in 5% nonfat milk in tris-buffered saline (TBS) for 1 h at room temperature, then probed with primary antibody diluted in 5% nonfat milk in TBS with 0.01% Tween-20 (TBST) overnight at 4 °C. Primary antibodies consisted of: rabbit anti-Fibronectin1 (abcam ab2413), mouse anti-Albumin (Millipore MAC115), rabbit anti-Vimentin (abcam ab271286), rabbit anti-TGFBI (also known as ßIG-H3 from Cell Signaling Technologies #2719, Danvers, MA, USA), rabbit anti-CD9 (Cell Signaling Technologies D801A), mouse anti-CD63 (Santa Cruz Biotech sc-5275, Dallas, TX, USA). Membranes were then washed three times with TBST then secondary antibodies conjugated to 680 LT or 800 CW flurophores (LiCor), diluted at 1:20,000 or 1:15,000 respectively in 5% nonfat milk in TBST were added and incubated for 1 h at room temperature. The membranes were washed again in TBST 3–5 times then visualized with the Odyssey CLx system (LiCor, Lincoln, NE, USA). Molecular weights were observed by the use of SeeBlue molecular weight standard (Thermo). Signal intensity was measured using Image Studio Acquisition Software system (LiCor) and statistical significance was measured using the Student’s *t*-test where noted.

### 2.8. Gene Ontology Analysis

Gene Ontology analysis was carried out as previously described [78] for proteins detected in coACCM by LC-MS/MS that were undetectable from unconditioned SFM, using the “GO Enrichment Analysis” tool at geneontology.org; date last accessed on 14 July 2022.

## 3. Results

### 3.1. Optimization of a Co-Cultured Amniotic Cell-Conditioned Media System

Amniotic fluid contains high levels of urea [79] and certain proteins, such as albumin [80] and immunoglobulin [81], while the relative levels of pro-growth and anti-inflammatory cytokines are lower. Moreover, AF donor-to-donor variability [46] suggests that standardized approaches such as biomanufacturing using only the cell type(s) of interest might serve to overcome these issues. We hypothesized that the co-culture of AECs and AFCs would maximize the production of regenerative factors while reducing the donor-specific variability observed with AF. To test this hypothesis, we first obtained AECs and AFCs and used immunostaining to determine whether these cells retain the multipotency biomarker SSEA4 after expansion for three passages (Figure 1A), consistent with previous reports [82]. We then mitotically inactivated the AECs and plated these as a “feeder layer” as is commonly used in embryonic stem cell culture with mouse embryonic fibroblasts (MEF) [83], or recently even AECs [84]. The expanded AFCs were plated on top of the inactivated AECs, allowed to attach for six hours, then serum-containing media was removed, the cells were thoroughly washed, and then we added two different types of serum-free media (SFM) and cultured the cells for 24 h then collected the conditioned media for analysis (Figure 1B). We found that SFM1 strongly stimulated the production of diverse proteins in AEC- and AFC-only media, and had an additive effect when the cells were co-cultured (Figure 1C). We concluded that SFM1 (heretofore referred to simply as SFM) is an optimal media in which a protein-rich coACCM can be generated within a 24 h timeframe.

To further characterize its components, we analyzed unconditioned SFM only, coACCM, AEC-only conditioned media, and AFC-only conditioned media by liquid chromatography tandem mass spectrometry (LC-MS/MS; see Supplementary Table S1). We observed proteins of note: Fibronectin 1 (FN1), Transforming Growth Factor-ß-Induced Protein (TGFBI), and Vimentin (VIM) found in coACCM that might elicit different responses in target cells/tissues (Supplementary Table S1). Clustering analysis of proteins identified with high confidence indicated the proteomes of coACCM and AFC-only conditioned media (with the exception of the AFC3 outlier) were more similar, and AEC-only conditioned media was less similar (Figure 1D). We next asked if any particular proteins that represented gene set annotations associated with certain cellular components, molecular functions, or biological processes were significantly enriched in coACCM compared to unconditioned SFM. We performed gene ontology (GO) analysis and found “extracellular exosome” to be the most enriched term (Figure 1E). Additional terms of interest that were significantly enriched include various cell adherence-associated terms, and cell signaling-based terms such as “NF-kappaB/NIK signaling”, “Wnt signaling…”, “tumor necrosis factor-mediated signaling”, among others (Figure 1E). Based on our LC-MS/MS findings (Supplementary Table S1) and known proteins present in AF, we compared the abundance of Albumin (ALB), FN1, TGFBI, and VIM in AF to coACCM by Western blot. In corroboration with the LC-MS/MS results, we readily detected FN1, TGFBI, and VIM proteins in coACCM (Figure 1F). Comparatively, ALB was readily detected in AF but undetectable in coACCM, and FN1 was significantly lower in AF than coACCM (*p* < 0.05 by Student’s *t*-test); TGFBI and VIM were undetectable in AF (Figure 1F). Based on these findings, we conclude that our optimized coACCM generates a rich proteome, but one that appears to be dissimilar to AF, and therefore may elicit different cellular responses.

### 3.2. coACCM Activates and AF Represses EMT

**Figure 2:**
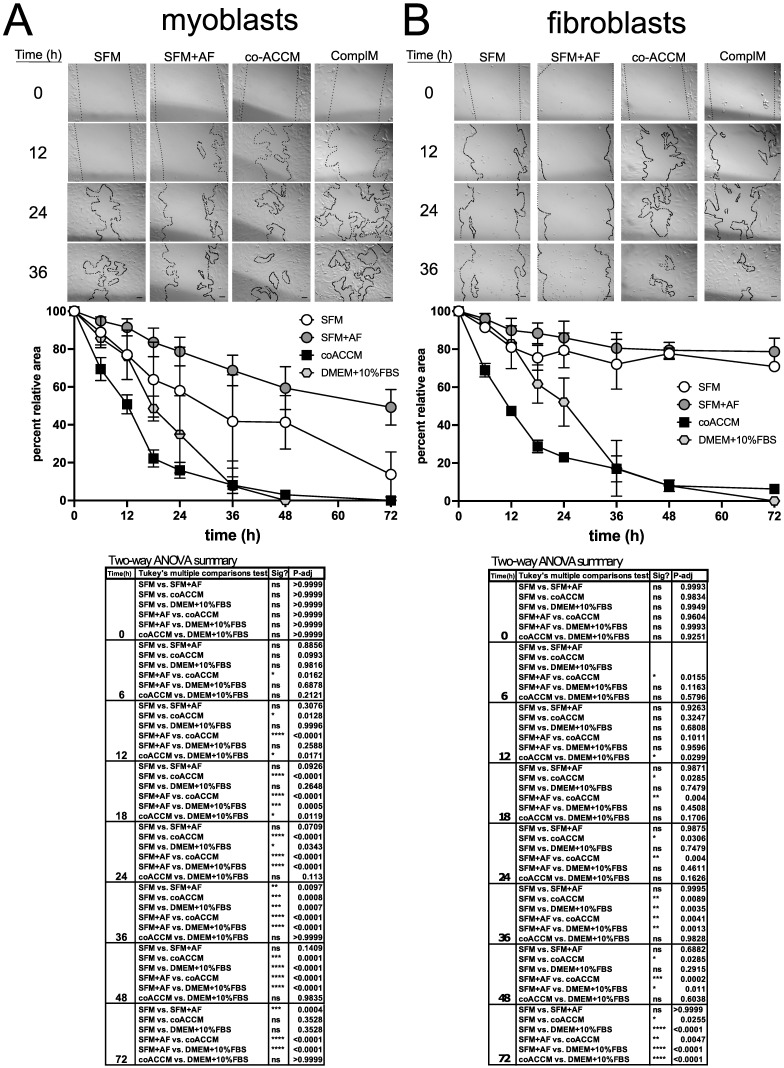
**Cell migration is activated by coACCM and repressed by AF.** Scratch test assays in C2C12 myoblasts (**A**) or MMM fibroblasts (**B**) cultured in SFM, SFM + 10% AF, co-ACCM, or complete media (complM) showing representative images from phase contrast microscopy at 0, 12, 24 and 36 h (*n* = 3 biological replicates). The dotted line “mask” defines the area devoid of cells. The graph below shows the mean percentage of area devoid of cells relative to time zero at the indicated time points, and error bars show the standard deviation. Below is the summary of the two-way ANOVA analyses with multiple comparisons (ns = not significant, * *p* < 0.05, ** *p* < 0.01, *** *p* < 0.001, **** *p* < 0.0001).

Since coACCM was rich in components associated with and capable of potentiating EMT, such as FN1 [85,86], Vimentin [87], and TGFBI [88], we hypothesized that it might activate this process. Similarly, full-term AF has been shown to promote AEC migration in vitro [51,52], but it is an open question as to how it might influence other cell types. We first tested this hypothesis using the scratch test cell migration assay, which is a functional assay that directly measures cell movement [89]. We used mouse C2C12 myoblasts [69] and MMM fibroblasts [70], which are both mesenchymal cell types, due to their broad relevance in the context of myofibroblast activation. The positive control for cell proliferation was complete media (DMEM with 10% fetal bovine serum (FBS)) and negative control was unconditioned SFM. Intriguingly, in both myoblasts and fibroblasts, coACCM was the most potent and rapid inducer of cell migration (Figure 2A,B). In contrast, the addition of 10% AF to SFM resulted in the most efficient repression of cell migration (Figure 2A,B). We conclude that coACCM significantly promotes, while AF significantly represses, myoblast and fibroblast migration (two-way ANOVA, upper bound *p* < 0.05).

**Figure 3:**
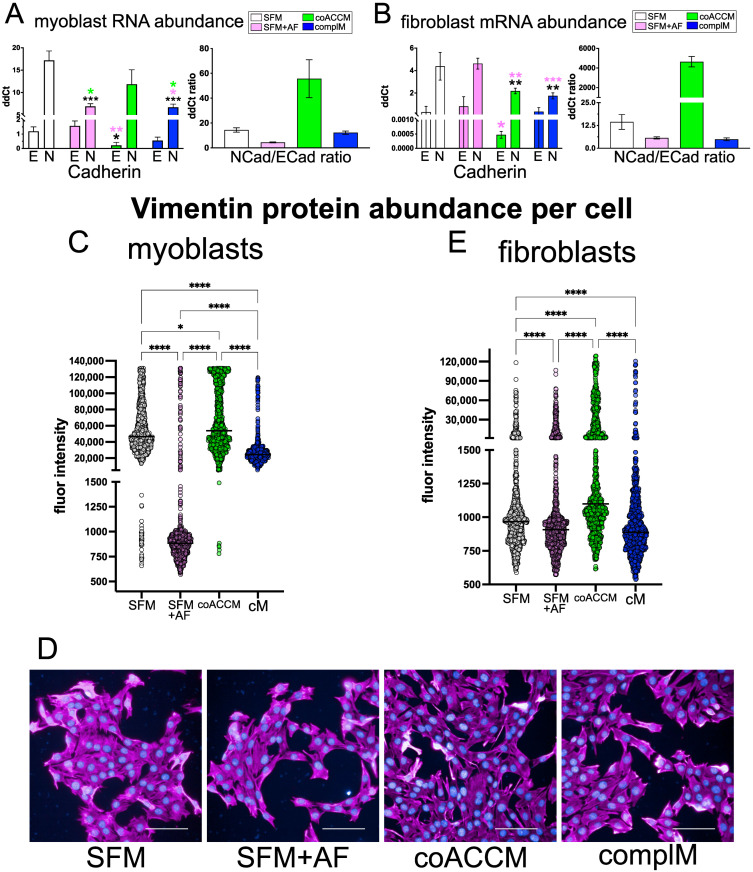
**coACCM activates EMT biomarker expression, while AF represses their levels.** (**A**) RT-qPCR measuring *N-Cadherin*, *E-Cadherin* or the ratio of *N-cadherin* to *E-cadherin* mRNA abundance from myoblast (**A**) or fibroblast (**B**) extracts following scratch test assay described above. The y-axis value indicates the mean ddCt calculated relative to *Hmbs* mRNA abundance (*n* = 3 biological replicates; * *p* < 0.05, ** *p* < 0.01, *** *p* < 0.001 by one-way ANOVA and is color-coded (significant relative to SFM is black, relative to AF is pink, and relative to coACCM is green); no statistical significance was measured for the ratio of *N-Cadherin* to *E-Cadherin* mRNA since a relative ratio is statistically incalculable but shown to indicate a change in magnitude. (**C**) High content screening analysis of indirect immunofluorescence staining for Vimentin protein in myoblasts in which fluorescence intensity per cell (*n* = 1000 per condition) was calculated for each media condition described above: SFM, SFM + AF, coACCM or complete media (cM); statistical significance was determined by Mann–Whitney U analysis; **** *p* < 0.0001. (**D**) Representative images from the analysis described in (**C**) above showing Vimentin (magenta) and DAPI (blue). Scale bar = 100 µm. (**E**) High content screening analysis as in (**C**) but in fibroblasts (**** *p* < 0.0001 by Mann–Whitney U).

We next asked if the addition of coACCM or AF elicited similar effects on EMT in these target cells at the molecular or cellular level. A common metric through which EMT/MET is assessed is the ratio of N-Cadherin (N-Cad) to E-Cadherin (E-Cad), where a higher ratio indicates a bias toward EMT, while a lower ratio favors MET [90]. At the conclusion of the scratch test assays, we extracted RNA from the cells and measured the relative levels of N-Cad and E-Cad and observed lower levels of the N-Cad:E-Cad ratio in myoblasts and fibroblasts treated with AF, but an increase in extracts from cells treated with coACCM (Figure 3A,B). We further investigated this with high content imaging by measuring Vimentin (which increases during and promotes EMT [91]) protein on a per-cell basis (*n* = 1000 cells per conditions after 72 h treatment). Intriguingly we found that the addition of AF to myoblasts was sufficient to significantly reduce Vimentin protein levels per cell, compared to all other conditions (**** *p* < 0.0001 by Mann–Whitney U; Figure 3C,D and Supplementary Table S2). Additionally, the highest Vimentin levels were observed in cell incubated with coACCM (Figure 3C,D). We observed a similar trend in fibroblasts, albeit to a lesser magnitude (Figure 3E). Taking these functional migratory, molecular, and cell-based results together, we conclude that coACCM potently activates EMT, while AF represses it.

### 3.3. coACCM Activates and AF Represses Inflammatory Biomarkers’ Abundance

**Figure 4:**
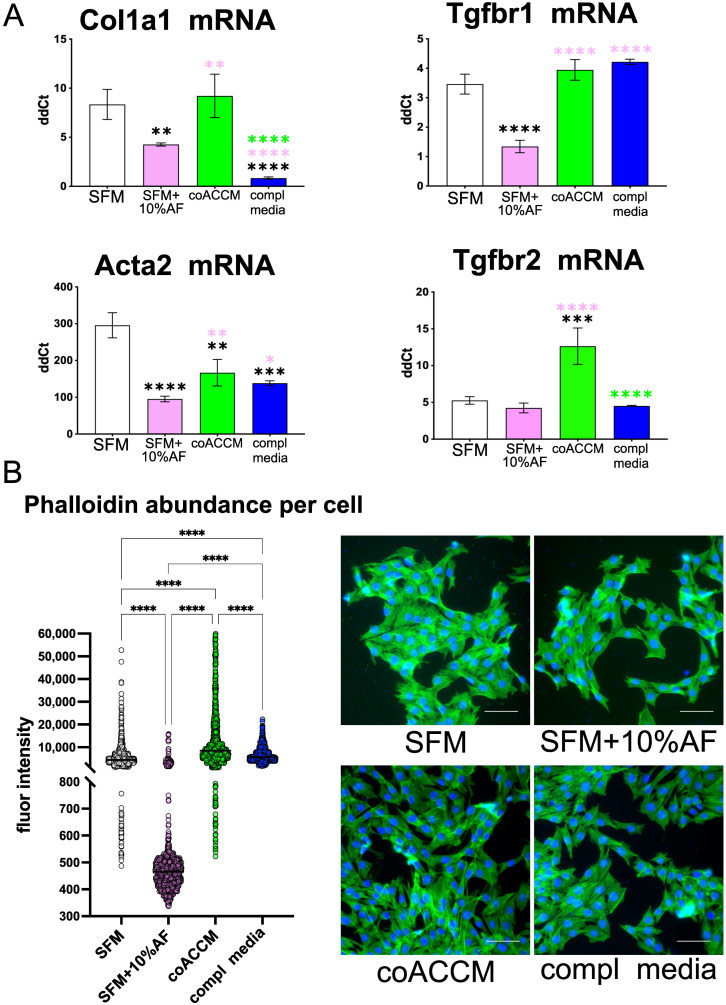
**coACCM promotes MFA-associated gene expression in myoblasts, while AF reduces it.** (**A**) RT-qPCR measuring *Col1a1*, *Tgfbr1*, *Acta2* and *Tgfbr2* mRNA extracted from C2C12 myoblasts cultured in biological triplicate in SFM, SFM + 10% AF, coACCM or complete media (compl media); * *p* < 0.05, ** *p* < 0.01, ***p < 0.001, and **** *p* < 0.0001 by one-way ANOVA and indicated by asterisks using the same color scheme described above. (**B**) High content screening analysis of phalloidin-stained (green) myoblasts’ fluorescence intensity per cell (left) with representative images shown on the right; scale bar = 100 µm; **** *p* < 0.0001 by Mann–Whitney U analysis.

Activating EMT is a critical early step in myofibroblast activation, and so we wondered if AF and coACCM would elicit comparable responses on inflammatory gene expression and cellular biomarkers. We measured mRNA production from *Col1a1* and *Acta2* in myoblast extracts following the 72 h treatment described above, and found the addition of 10% AF to SFM resulted in a significant reduction in both (** *p* < 0.01 and **** *p* < 0.0001, respectively, by one-way ANOVA), when compared to SFM alone (Figure 4A). The levels of *Col1a1* and *Acta2* mRNA in extracts from coACCM-treated myoblasts were also significantly higher than those observed from AF-treated myoblasts (both ** *p* < 0.01 by one-way ANOVA; Figure 4A). The Transforming Growth Factor ß (TGF-ß) pathway is also a mediator and effector of fibrosis-associated conditions, including MFA [92,93]. In the presence of TGF-ß (and therefore active TGF-ß signaling), the *Tgfbr1* and *Tgfbr2* mRNA levels increase [94], and thus we used their mRNA levels as a proxy for TGF-ß activation. Myoblasts treated with AF showed lower levels of *Tgfbr1* and *Tgfbr2* transcripts compared to those treated with coACCM (Figure 4A), suggesting that AF can reduce TGF-ß activation. When we made the same comparisons as above, but in fibroblasts, we observed similar results with one exception: the addition of AF resulted in higher levels of *Acta2* mRNA (Supplementary Figure S2A). Finally, we quantified phalloidin staining per cell as a measure of stress fiber formation, and observed significantly lower levels in myoblasts and fibroblasts when comparing cells treated with AF to coACCM (Figure 4B,C and Figure S2B, respectively; **** *p* < 0.0001 by Mann–Whitney U; see also Supplementary Table S2). We also note an overall more diffuse distribution of the phalloidin signal in myoblasts treated with AF compared to a more rod-like concentrated appearance observed in those treated with coACCM (Figure 4C), further suggesting the formation of stress fibers in the presence of coACCM. Together these data indicate that AF reduces the levels of MFA molecular and cellular biomarkers while coACCM increases them.

### 3.4. AF Prevents TGF-ß-Induced Myofibroblast Activation

**Figure 5:**
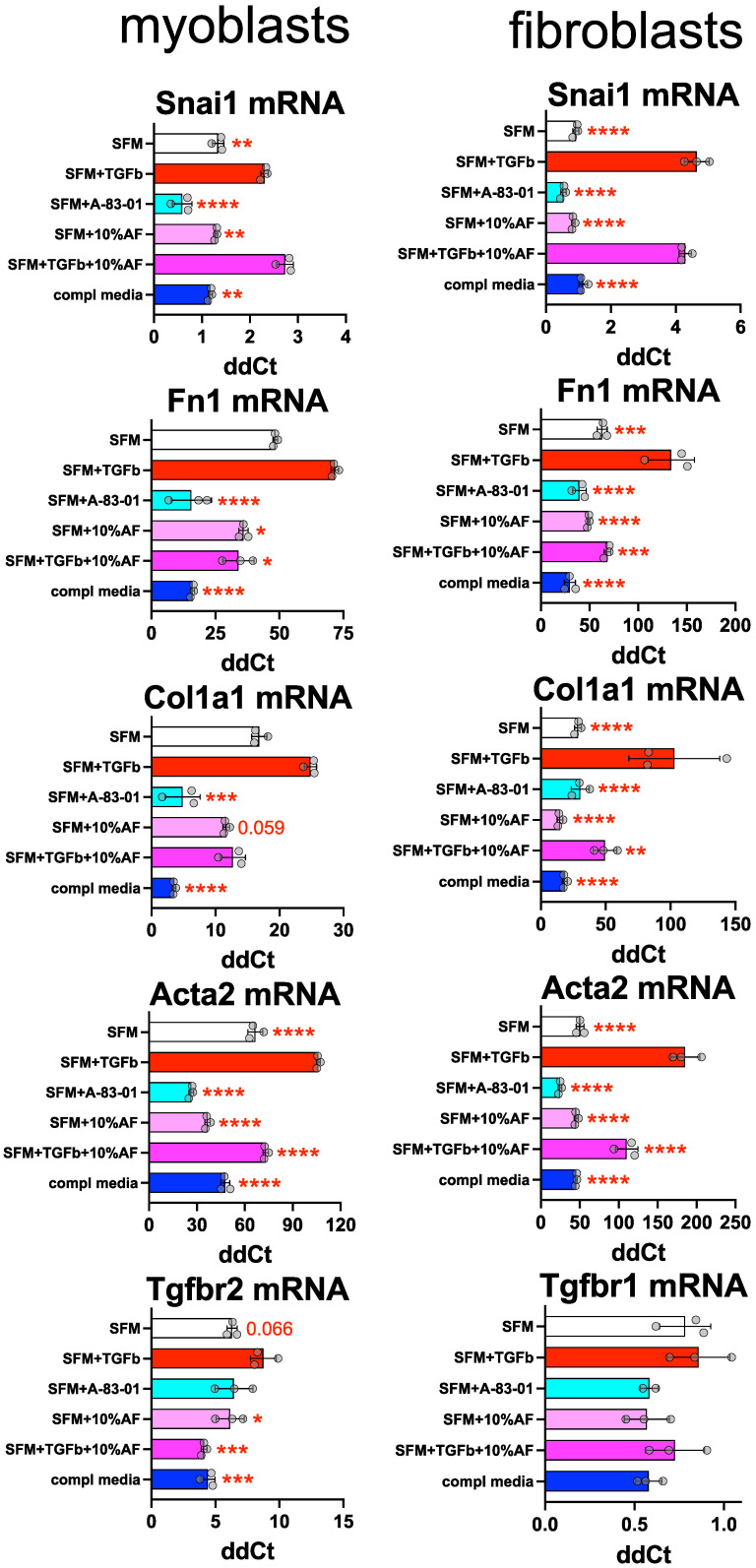
**AF represses TGF-ß-activated EMT and MFA.** RT-qPCR of extracts from myoblasts (left) or fibroblasts, measuring mRNA from *Snail1*, *Fn1*, *Col1a1*, *Acta2* and *Tgfbr2* relative to *Hmbs* mRNA. The cells were cultured in biological triplicate using the following media conditions: SFM, SFM + 5 ng/mL TGF-ß, SFM + A-83-01 (a TGF-ß inhibitor), SFM + 10% AF, SFM + TGF-ß and 10% AF or complete media for 24 h then the RNA was extracted and analyzed. Error bars represent the SD from the mean ddCt values (* *p* < 0.05, ** *p* < 0.01, *** *p* < 0.001, **** *p* < 0.0001 by one-way ANOVA are shown (and in some cases the *p*-value) compared to the SFM + 5 ng/mL TGF-ß condition).

Given that AF prevents EMT and lowers MFA biomarkers, while coACCM stimulates these, we chose to focus on if and how AF can prevent MFA. We hypothesized that AF could block TGF-ß stimulated MFA, since we observed reduced levels of *Tgfbr2* or *Tgfbr1* mRNA levels after 72 h treatment of myoblasts or fibroblasts, respectively (Figure 4A and Figure S4A). To test this, we incubated cells in SFM, SFM with 5 ng/mL TGF-ß, SFM with 1 µM A-83-01 (TGF-ß type I receptor inhibitor and inhibitor of EMT [95]), SFM with 10% AF, SFM with 5 ng/mL TGF-ß and 10% AF, or complete media for 24 h then measured EMT and MFA biomarkers’ mRNA abundance. As expected, the addition of TGF-ß to SFM resulted in increased mRNA abundance of *Snai1*, *Fn1*, *Col1a1*, *Acta2*, and *Tgfbr2* or *Tgfbr1* in myoblasts or fibroblasts, respectively (Figure 5), consistent with the activation of EMT and MFA. The addition of A-83-01 abrogated TGF-ß-induced increases in the abundance of each of these gene products, as did the addition of 10% AF (Figure 5). Intriguingly, adding 10% AF to SFM that contained 5 ng/mL TGF-ß also had protective effects against the induction of *Fn1*, *Col1a1*, and *Acta2* in both myoblasts and fibroblasts (Figure 5), and in myoblasts there was a synergy between the inclusion of AF and TGF-ß on the repression of *Tgfbr2* mRNA levels (Figure 5; compare reduction with SFM + 10%AF at * *p* < 0.05 to greater magnitude reduction with SFM + TGF-ß + 10%AF at *** *p* < 0.001 by one-way ANOVA). Therefore, AF reduces basal and TGF-ß-activated EMT and MFA in both myoblasts and fibroblasts.

### 3.5. AF-EVs Are Necessary and Sufficient for EMT and MFA

**Figure 6:**
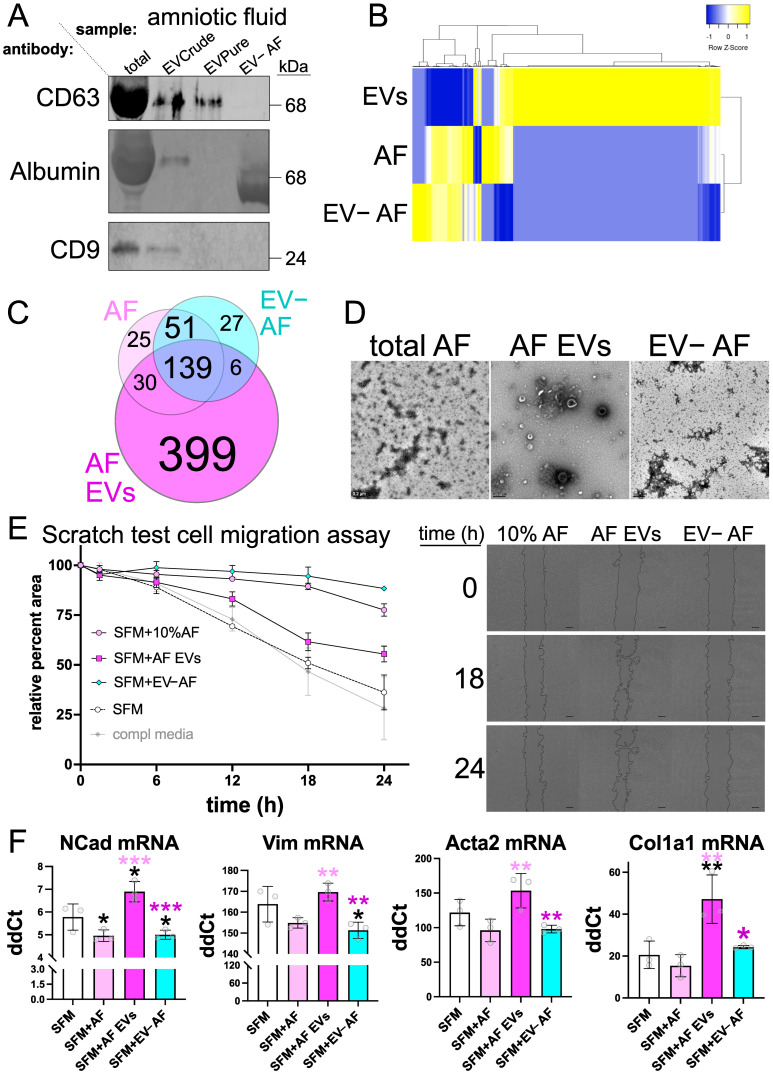
**AF EVs promote EMT and MFA while EV-depleted AF inhibits.** (**A**) Western blot of 1 µg total AF, 1 µg of crude extract from EV enrichment, an equal volume of purified EVs, and 1 µg of EV-depleted AF probed with anti-CD63, anti-Albumin or anti-CD9 antibodies (see also Supplementary Figure S4 for uncropped Western blots and sequential probing scheme; we note that the signal shown for CD63 in total AF is likely an artifact from highly abundant Albumin protein). (**B**) Proteomic profile of biological replicate 2 (which had the deepest proteomics coverage) showing LC-MS/MS-detected protein abundance from purified AF EVs (EVs), total AF (AF), or EV-depleted AF (EV-AF), and clustered by similarity. (**C**) Venn diagram depicting the number of peptide/proteins from AF, AF EVs, or EV-AF identified by LC-MS/MS overlap or are distinct between each sample. (**D**) Transmission electron microscopy analysis of total AF, AF EVs and EV-depleted AF (EV-AF) purified by ultracentrifugation; scale bar 0.2 μm. (**E**) Scratch test assay quantification of myoblast migration relative to time zero (left) or representative phase contrast images used to derive these values (right); scale bar = 200 µm. See Table 1 for statistical analyses summary. (**F**) RT-qPCR of RNA extracted after the conclusion of a scratch test assay from myoblasts treated in (**E**) above; * *p* < 0.05, ** *p* < 0.01, *** *p* < 0.001 by one-way ANOVA); the data shown in Figure 6E,F used AF from a single donor, independent from those data shown in Supplementary Figure S3C.

**Figure 7:**
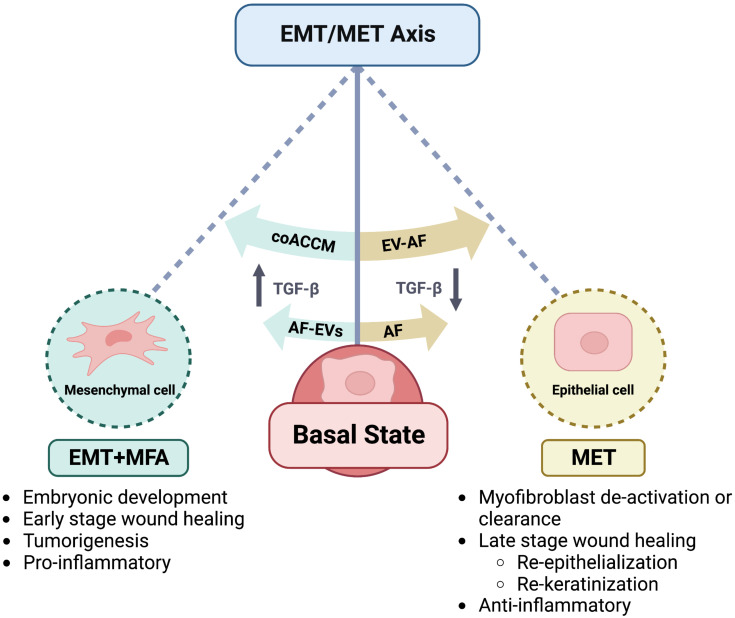
**Model and Summary/Conclusion of findings.** We use a pendulum as an analogy to represent the EMT/MET axis. The middle represents the basal state of a cell which can be influenced toward EMT and MFA (left) by the addition of TGF-ß, coACCM, or AF EVs), or away from EMT and MFA, toward MET and reduction in MFA (right) by the addition of TGF-ß inhibitor A-83-01, total AF, or EV-AF. EMT is normally observed during embryonic development, and EMT and MFA are normally observed during the early stages of wound healing, and can drive tumorigenesis and pro-inflammatory responses. In contrast, induction of MET and inhibition of MFA and/or myofibroblast clearance is associated with the late stages of wound healing and anti-inflammatory effects.

AF is a rich source of EVs, and our proteomics analysis of coACCM indicated a significant enrichment of exosome/EV-related terms (Figure 1). Therefore, we asked if EVs or EV-depleted coACCM or AF showed any differential effects on cell migration/EMT or MFA. We used a commercially available kit to purify EVs from coACCM then validated this by Western blotting and found an enrichment of CD63 and depletion of TGFBI in the purified EV fraction (Supplementary Figure S3A). We compared the effect of total coACCM, the same number of purified EVs from coACCM added back to SFM, and EV-depleted coACCM, on cell migration using the scratch test assay and found no significant differences (Supplementary Figure S3B). We used an identical approach with AF, and also validated EV purification by Western blot (Figure 6A) and LC-MS/MS (Supplementary Table S3), and observed that, remarkably, EV-depleted AF (EV- AF) potently repressed cell migration (Supplementary Figure S3C). In addition, there was a concomitant reduction in the mRNA levels of N-Cad/E-Cad ratio, Vimentin, Acta2 and Col1a1 when EV- AF was added, and an increase in these upon the addition of AF EVs (Supplementary Figure S3D). Furthermore, comparing the LC-MS/MS data indicated that total AF and EV- AF were more similar while AF EVs had a dissimilar proteomic profile (Figure 6B) with many more unique peptides/proteins observed in the AF EVs (Figure 6C). We sought to cross-validate these intriguing results using AF from a different donor and another method of EV purification, and so used ultracentrifugation to do so. Analysis of the total AF, AF EVs, and EV- AF by transmission electron microscopy indicated a successful purification of EVs of the expected size range (~50–250 nm) and depletion of EVs from EV- AF (Figure 6D). Moreover, instead of adding back an equal amount of EVs to SFM, this time we added back an equal amount of EV protein to that found in SFM with 10% AF, and likewise for EV- AF, and performed the scratch test assay as previously. We again observed the strongest repression of cell migration with the addition of EV- AF, an intermediate level of repression with SFM + 10% AF, and more migration upon the addition of AF EVs, albeit not to the extent observed with SFM (Figure 6E and Table 1). Moreover, we found a significant increase in N-Cad, Vimentin, Acta2, and Col1a1 mRNA abundance upon the addition of AF EVs, which was lowered when EV- AF was added (Figure 6F), consistent with our previous results (Supplementary Figure S3D). Altogether, these data indicate that AF EVs are necessary and sufficient for cell migration/EMT and MFA, and suggest that different formulations of coACCM or fractions of AF could be useful biologics in various precision medicine settings (Figure 7 and see below).

## 4. Discussion

Recent studies have highlighted the utility of AF [27,30,34,35,36,37] and AFC- or AEC-conditioned media [66,67], but the underlying mechanisms through which their therapeutic effects are mediated have received less study. Here we show that co-culturing AECs and AFCs in SFM1 produces a more diverse proteome, and that the resulting coACCM activates EMT and MFA, while AF inhibits these processes. Intriguingly, the fractionation of AF into EV-enriched or EV-depleted fractions also has opposing effects on these processes whereby AF EVs promote EMT and MFA, while EV-depleted AF inhibits EMT and MFA. This opens the possibility of their usefulness, in combination or as a stand-alone, in a variety of clinical and biotechnological applications.

EMT and MFA are important physiological responses that maintain tissue homeostasis, but can have deleterious effects if not resolved. For example, in wound healing, MFA and EMT are required early steps to initiate the cascade of events required to begin healing, but their persistence can lead to non-healing wounds or worsen scarring [96]. In such a case, one might want to first treat a non-healing wound with coACCM or AF EVs to re-initiate the inflammatory and/or proliferative states of wound healing. Following that, treatment with total AF would likely initiate the transition toward MET and reduce MFA to stimulate the transition from the proliferative state toward the remodeling state and promote wound closure. Finally, a later treatment using EV-AF could promote re-epithelialization and re-keratinization to conclude the remodeling state. Currently, AF is being used to successfully treat complex, non-healing wounds in the clinic [19,20], but patients affected by diabetic foot ulcers or venous stasis ulcers often still face limb amputation as their final option. Future studies testing this multipronged approach with coACCM and fractionated AF are required to determine if these novel regenerative medicine solutions can improve outcomes in complex wound treatment. Additionally, investigations into musculoskeletal disease and other conditions in which persistent inflammation is the underlying etiology, are necessary to further dissect the scope of utility of these novel regenerative solutions.

## 5. Conclusions

We find here that coACCM and AF elicit different functional responses in the contexts of EMT and MFA, in myoblasts and fibroblasts. These cell types are relevant in a variety of disease and developmental contexts. We also note here that one limitation of the current study is that it was conducted using in vitro cell culture as the model, and so follow-up studies using ex vivo tissue/organ models or animal models should be considered. Given the widespread involvement of myofibroblasts in a plethora of disease processes, further fundamental and translational studies are required to determine the full potential of these innovative reagents.

## 6. Patents

T.C.B. and W.S.F. are holders of patents WO/2019/040790A1, PCT/US2018/47818 and WO/2020/176801, PCT/US2020/020229 involving the subject matter contained herein.

## Figures and Tables

**Table 1:** Two-way ANOVA of scratch test assay data from Figure 6E. ns = not significant, * *p* < 0.05, ** *p* < 0.01, *** *p* < 0.001, **** *p* < 0.0001.

Time (h)	Tukey’s Multiple Comparisons Test	Sig?	P-adj
1.5	SFM+10%AF vs. SFM+AF EVs	ns	0.4763
	SFM+10%AF vs. SFM+EV-AF	ns	0.3416
	SFM+10%AF vs. SFM	ns	>0.9999
	SFM+AF EVs vs. SFM+EV-AF	ns	0.9973
	SFM+AF EVs vs. SFM	ns	0.4209
	SFM+EV-AF vs. SFM	ns	0.1771
6	SFM+10%AF vs. SFM+AF EVs	ns	0.2842
	SFM+10%AF vs. SFM+EV-AF	ns	0.4644
	SFM+10%AF vs. SFM	ns	0.1466
	SFM+AF EVs vs. SFM+EV-AF	ns	0.1034
	SFM+AF EVs vs. SFM	ns	0.7403
	SFM+EV-AF vs. SFM	ns	0.0578
12	SFM+10%AF vs. SFM+AF EVs	ns	0.0955
	SFM+10%AF vs. SFM+EV-AF	ns	0.3642
	SFM+10%AF vs. SFM	****	<0.0001
	SFM+AF EVs vs. SFM+EV-AF	*	0.0252
	SFM+AF EVs vs. SFM	*	0.0491
	SFM+EV-AF vs. SFM	**	0.0072
18	SFM+10%AF vs. SFM+AF EVs	*	0.0117
	SFM+10%AF vs. SFM+EV-AF	ns	0.4166
	SFM+10%AF vs. SFM	***	0.0007
	SFM+AF EVs vs. SFM+EV-AF	**	0.003
	SFM+AF EVs vs. SFM	ns	0.0988
	SFM+EV-AF vs. SFM	**	0.0013
24	SFM+10%AF vs. SFM+AF EVs	**	0.007
	SFM+10%AF vs. SFM+EV-AF	*	0.045
	SFM+10%AF vs. SFM	*	0.0233
	SFM+AF EVs vs. SFM+EV-AF	**	0.0072
	SFM+AF EVs vs. SFM	ns	0.1241
	SFM+EV-AF vs. SFM	*	0.021

## Data Availability

Raw data from LC-MS/MS can be found at MassIVE under accession number MSV000090160.

## Supplementary Materials

The following supporting information can be downloaded at: https://www.mdpi.com/article/10.3390/biomedicines10092189/s1, Supplementary materials associated with this manuscript include Supplementary Figure S1: uncropped gel and western blot to accompany Figure 1, Figure S2: data supplemental to Figure 4, Figure S3: data supplemental to Figure 6, and Figure S4: uncropped western blots to accompany Figure 6; Supplementary Table S1: LC-MS/MS data and GO analysis pertaining to results described in Figure 1; Table S2: raw data from Opera Phenix analysis pertaining to the results described in Figure 3C,D,E, Figure 4B, and Figure S4B; Table S3: LC-MS/MS data pertaining to the results described in Figure 6.
