# Supplementary material for: Comparative Analysis of Co-Cultured Amniotic Cell-Conditioned Media with Cell-Free Amniotic Fluid Reveals Differential Effects on Epithelial–Mesenchymal Transition and Myofibroblast Activation
Source: Biomedicines. 2022 Sep 5;10(9):2189. doi: 10.3390/biomedicines10092189 (PMC9495976; doi:10.3390/biomedicines10092189)
Supplement: Supplementary file 1 [file biomedicines-10-02189-s001.zip › biomedicines-1898049-figures.pdf]

SUPPLEMENTAL FIGURE S1

A. Uncropped silver-stained SDS-PAGE

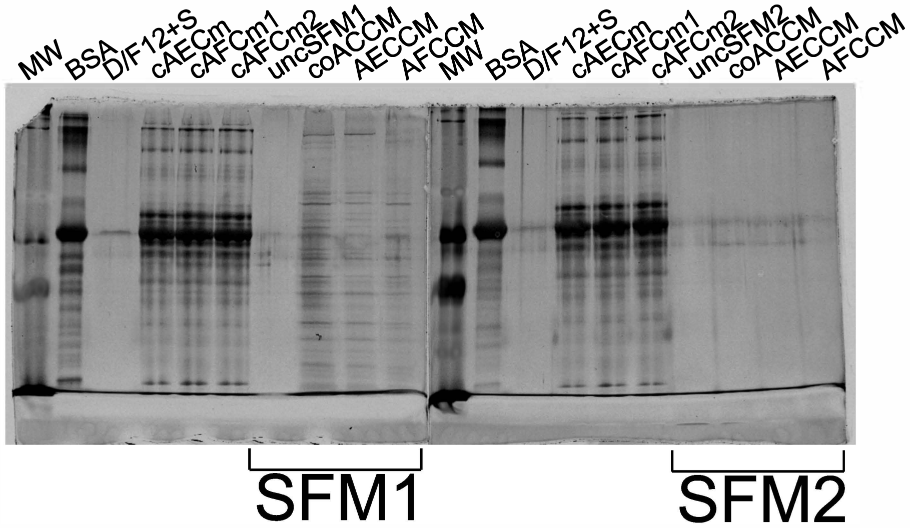

B. Uncropped western blots

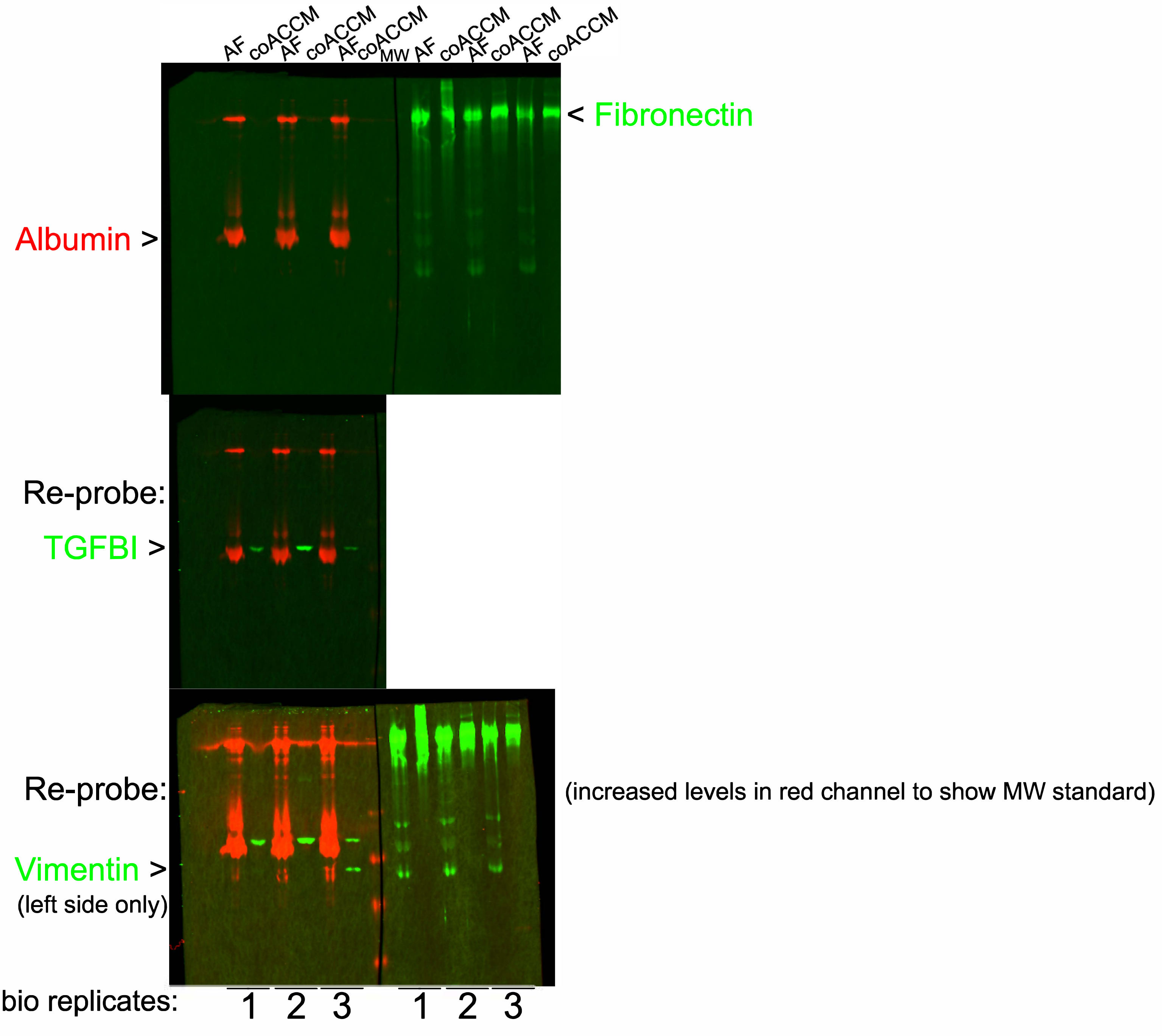

Supplemental Figure S1. Raw, unedited SDS-PAGE and Western Blot Data for Main Figure 1. Uncropped silver-stained SDS-PAGE profile of SFM1 and SFM2 in different media corresponding to Figure 1C. Uncropped western blot corresponding to Figure 1F (b) probing for albumin (*red*) followed by TGFBI (*green*) and Vimentin (*green*) (left) and Fibronectin followed by Vimentin (*right*).

SUPPLEMENTAL FIGURE S2

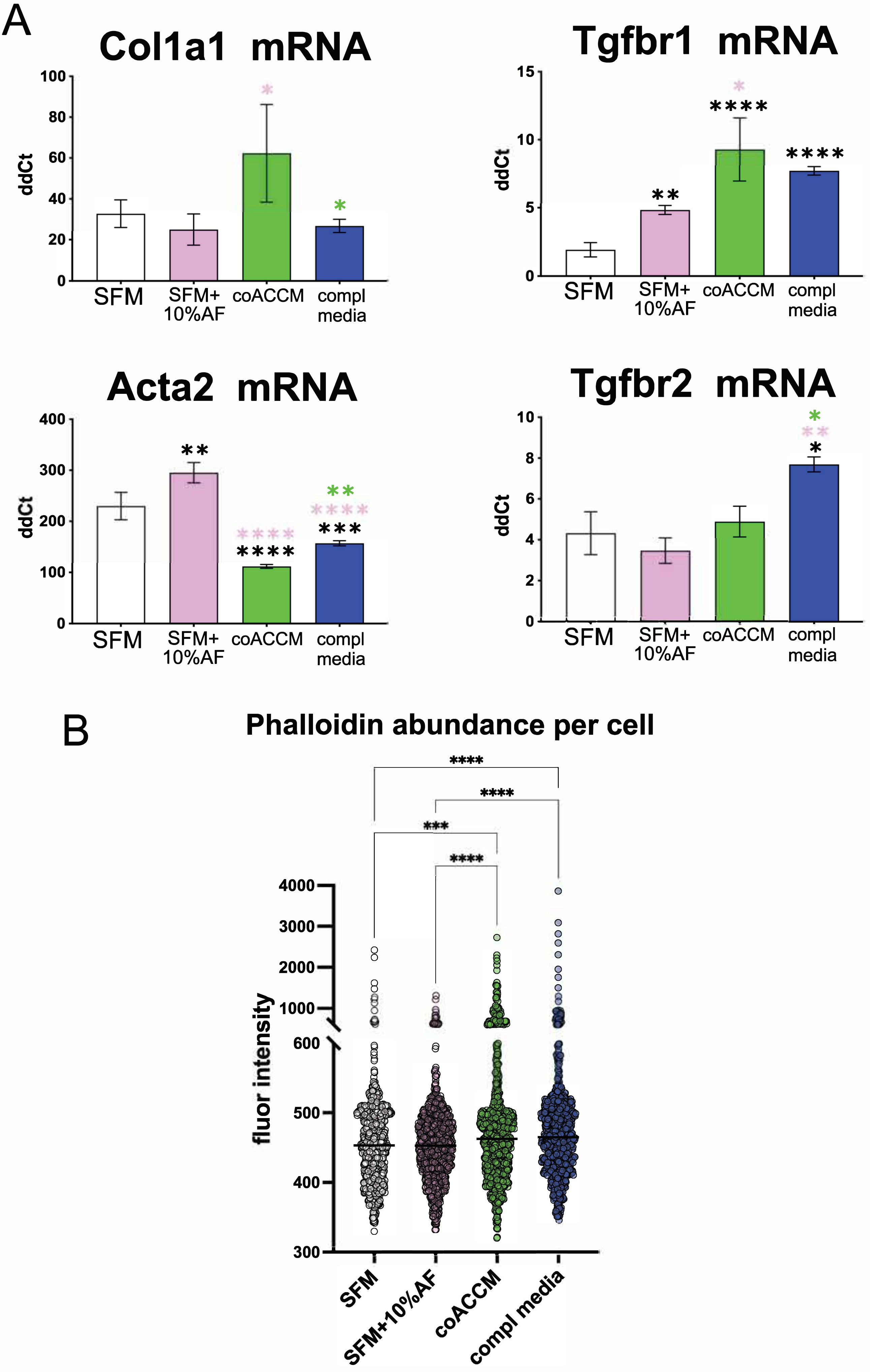

Supplemental Figure S2: coACCM promotes MFA-associated gene expression in fibroblasts, while AF reduces it. (a) RT-qPCR analysis as described above in Fig 4A, but using fibroblast RNA extracts. (b) High content screening analysis as described above in Fig 4B but using fibroblasts; $^{***}P < 0.001$, $^{****}P < 0.0001$ by Mann-Whitney U analysis.

SUPPLEMENTAL FIGURE S3

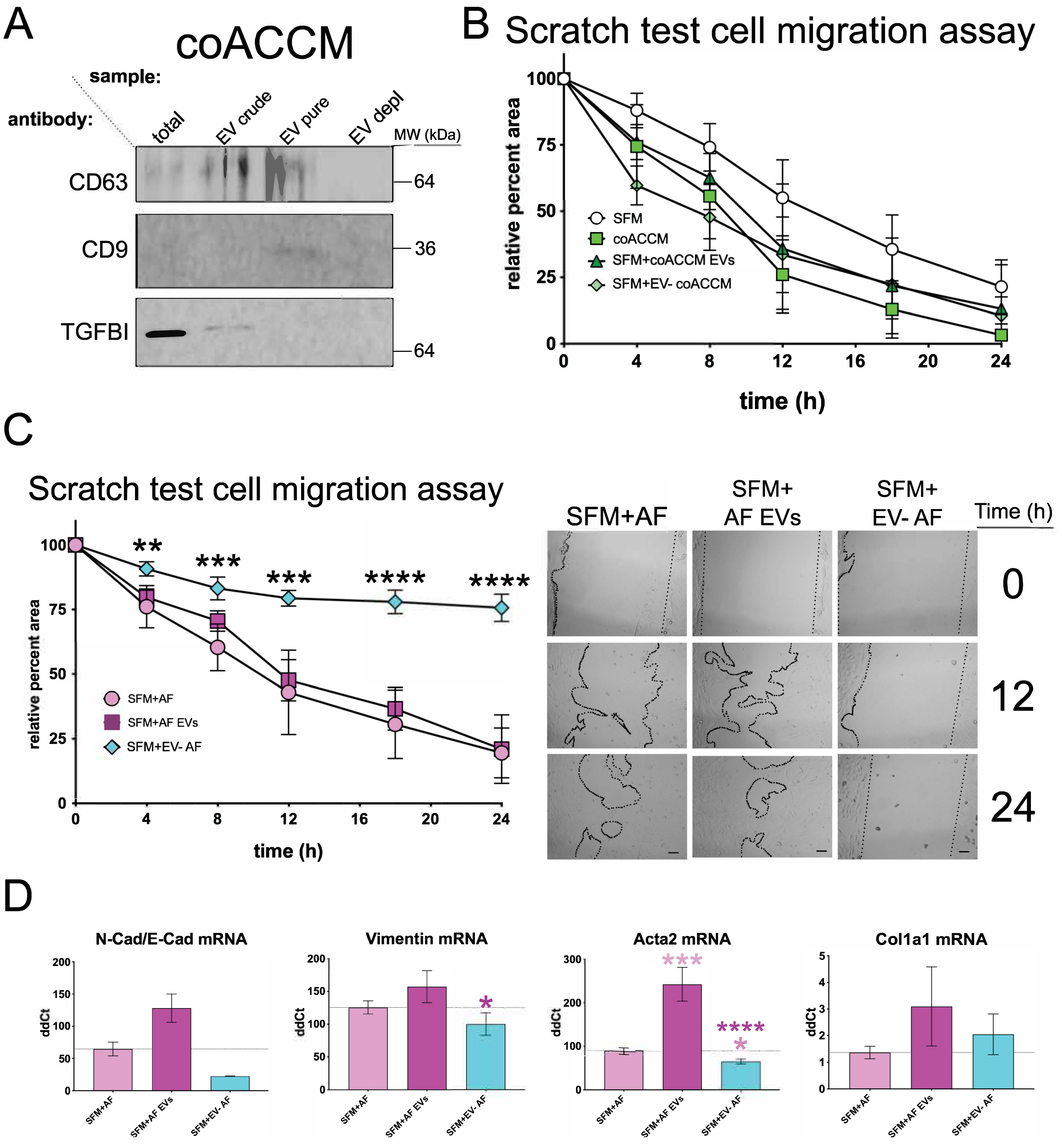

Supplemental Figure S3: AF EVs promote EMT and MFA while EV-depleted AF inhibits. (a) Western blot as described in Fig 6A but for coACCM EV purification; membranes were probed with antibodies for CD63, CD9 and TGFBI; MW in kDa is shown on the right. (b) Relative myoblast migration from scratch test assay for cells cultured in SFM, coACCM, SFM plus coACCM EVs (SFM + coACCM EVs), or EV-depleted coACCM (EV- coACCM); measurements were conducted in biological triplicate with mean values shown, and error bars denoting the standard deviation; no statistical significance was observed when comparing groups by two-way ANOVA. (c) Scratch assay quantification (*left*) and representative phase contrast microscopy images (right) of migration relative to time zero in myoblasts cultured in biological triplicate in SFM + 10% AF (SFM+AF), SFM + an equal number of AF EVs as that used in SFM+10%AF, or SFM + 10% EV- AF; $^{**}P < 0.01$, $^{***}P < 0.001$, $^{****}P < 0.0001$ by two-way ANOVA for EV- AF compared to each other sample; this used AF pooled from three independent donors as described in the proteomics analysis (Supplemental Table S2). (d) RT-qPCR of myoblast RNA extracted after the conclusion of the scratch test assay described in Supplemental Fig 6C, described above; $^{*}P < 0.05$, $^{***}P < 0.001$, $^{****}P < 0.0001$ by one-way ANOVA using the color scheme described previously.

SUPPLEMENTARY FIGURE S4

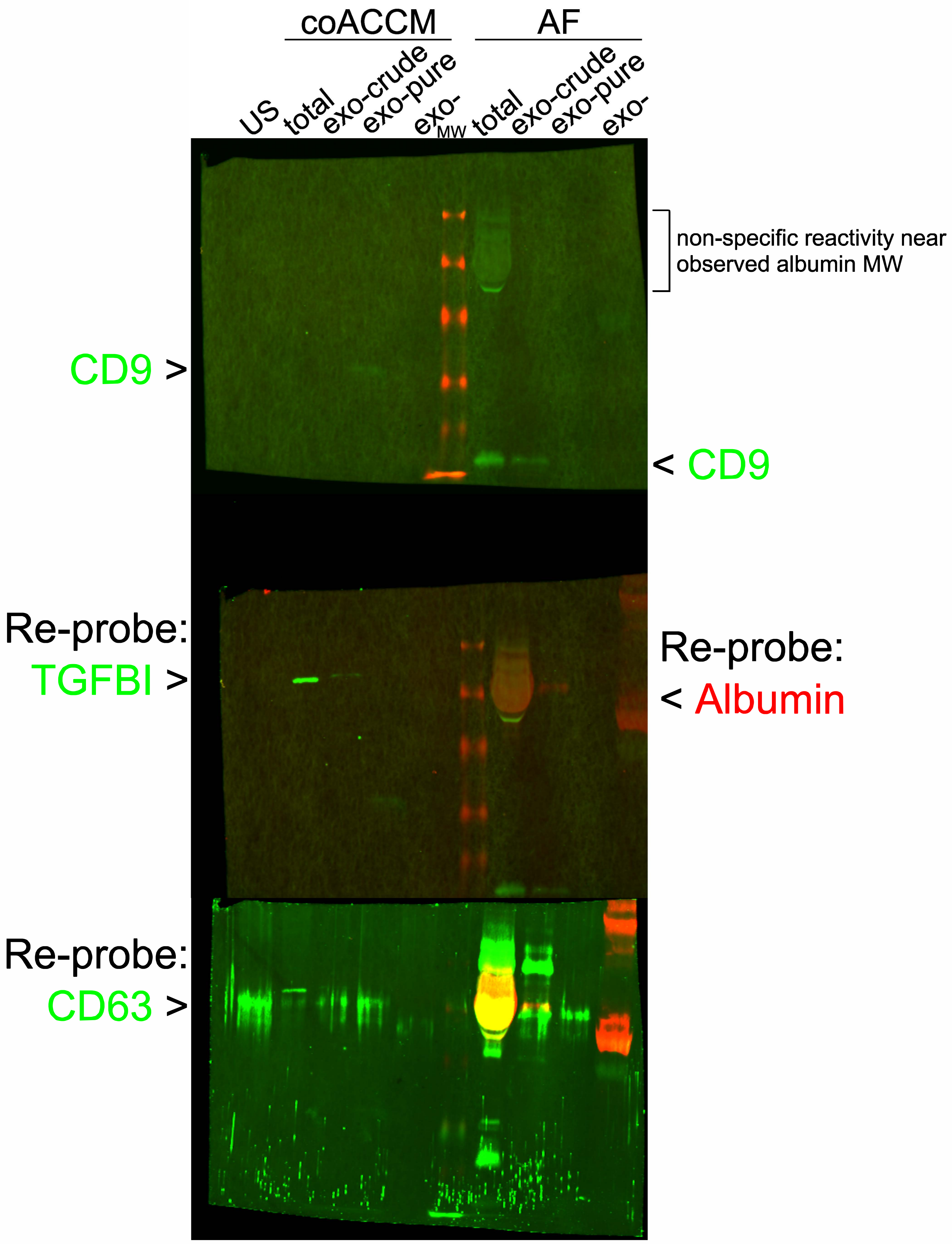

Supplemental Figure S4: Uncropped western blots from those shown in Figure 6 and Supplemental Figure 6 data corresponding to Western Blots in Supplemental Figure 6. See above and note that the reported observed molecular weights for CD9 are 24 and 35 kDa.
